# LncRNA H19 confers chemoresistance in ERα-positive breast cancer through epigenetic silencing of the pro-apoptotic gene BIK

**DOI:** 10.18632/oncotarget.13263

**Published:** 2016-11-10

**Authors:** Xinxin Si, Ruochen Zang, Erbao Zhang, Yue Liu, Xiao Shi, Ershao Zhang, Lipei Shao, Andi Li, Nan Yang, Xiao Han, Beijing Pan, Zhihong Zhang, Luan Sun, Yujie Sun

**Affiliations:** ^1^ Key Laboratory of Human Functional Genomics of Jiangsu Province, Nanjing Medical University, Nanjing, Jiangsu, China; ^2^ Collaborative Innovation Center for Cancer Medicine, Jiangsu Key Lab of Cancer Biomarkers, Prevention and Treatment, Nanjing Medical University, Nanjing, Jiangsu, China; ^3^ State Key Laboratory of Reproductive Medicine, Nanjing Medical University, Nanjing, Jiangsu, China; ^4^ Department of Cell Biology, Nanjing Medical University, Nanjing, Jiangsu, China; ^5^ Department of Biochemistry and Molecular Biology, Nanjing Medical University, Nanjing, Jiangsu, China; ^6^ Department of Pathology, First Affiliated Hospital of Nanjing Medical University, Nanjing, Jiangsu, China

**Keywords:** breast cancer, chemoresistance, lncRNA H19, apoptosis, estrogen receptor

## Abstract

Breast cancer is a common malignancy in women. Acquisition of drug resistance is one of the main obstacles encountered in breast cancer therapy. Long non-coding RNA (lncRNA) has been demonstrated to play vital roles in both development and tumorigenesis. However, the relationship between lncRNAs and the development of chemoresistance is not well established. In the present study, the high expression of lncRNA H19 was identified as a powerful factor associated with paclitaxel (PTX) resistance in ERα-positive breast cancer cells, but not in ERα-negative breast cancer cells. LncRNA H19 attenuated cell apoptosis in response to PTX treatment by inhibiting transcription of pro-apoptotic genes BIK and NOXA. H19 was further confirmed to suppress the promoter activity of BIK by recruiting EZH2 and by trimethylating the histone H3 at lysine 27. Interestingly, our data showed that lncRNA H19 was one of the downstream target molecules of ERα. Altered ERα expression may therefore change H19 levels to modulate the apoptosis response to chemotherapy in breast cancer cells. Our data suggest that the ERα-H19-BIK signaling axis plays an important role in promoting chemoresistance.

## INTRODUCTION

Breast cancer is a common malignancy in women around the world. Chemotherapy has been an effective treatment plan for most breast cancer patients, however, the emergence of chemoresistance now severely restrains the efficacy of this therapy. One hallmark of cancer is the disruption of apoptosis, which facilitates both tumorigenesis and chemoresistance. Consequently, most anticancer agents kill tumor cells by activating apoptotic pathways, but how this activation is circumvented in chemoresistant tumors is poorly understood. Improvements in cancer treatments therefore require a greater understanding of the molecular events that either make tumors susceptible to drug-induced apoptosis or allow them to evade apoptotic death.

Long non-coding RNA (lncRNA) is a type of non-coding transcript [1, 2]. Increasing evidence supports a vital role for lncRNA in tumor formation. It could regulate multiple cancer-associated signaling pathways involved in cell cycle, cell proliferation, apoptosis, and migration [3–8]. The H19 gene, which transcribes a long non-coding RNA, is a maternally expressed imprinted gene that plays a vital role in mammalian development [9–11]. H19 is highly expressed in a majority of human cancers, including breast cancer, colorectal cancer, hepatocellular carcinoma, and gastric cancer [12–15], and overexpression of H19 is often correlated with poor prognosis of tumor patients [16, 17]. Despite its importance, H19 expression has been poorly studied in terms of cell apoptosis and chemoresistance. Our research has revealed that high expression of H19 in breast cancer attenuates cell apoptosis normally seen in response to chemotherapy. Other studies have reported that H19 promotes the progression and metastasis of tumors in different ways, by serving as a miRNA sponge, by altering DNA methylation, and by controlling mRNA decay [10, 18, 19]. In the present paper, we have demonstrated an epigenetic inhibition of pro-apoptotic gene BIK by the association of H19 with the histone methyltransferase EZH2. This is the first confirmation of a role for H19 in breast cancer drug resistance.

The progression of breast cancer is regulated at least in part by the estrogen receptor α (ERα). About 65% of the human breast cancers are estrogen dependent and express ERα [20]. Accumulating data from clinical trials now suggest an involvement of ERα in the sensitivity of breast cancer cells to chemotherapeutic agents. For example, patients with ERα-positive breast cancers reportedly gain little benefit from the administration of PTX [21, 22]. Improving the efficacy of chemotherapy as a breast cancer treatment therefore requires a better understanding of its underlying molecular mechanisms. We have shown here that the lncRNA H19 is one of the downstream molecules of ERα in breast cancer cells, and that upregulation of H19 by ERα modulates the apoptosis response to chemotherapy in breast cancer cells. Based on these findings, we propose that the ERα-H19-BIK signaling axis is involved in the promotion of PTX resistance.

## RESULTS

### The expression level of lncRNA H19 was positively correlated with PTX resistance in ERα-positive breast cancer cells

**Figure 1:**
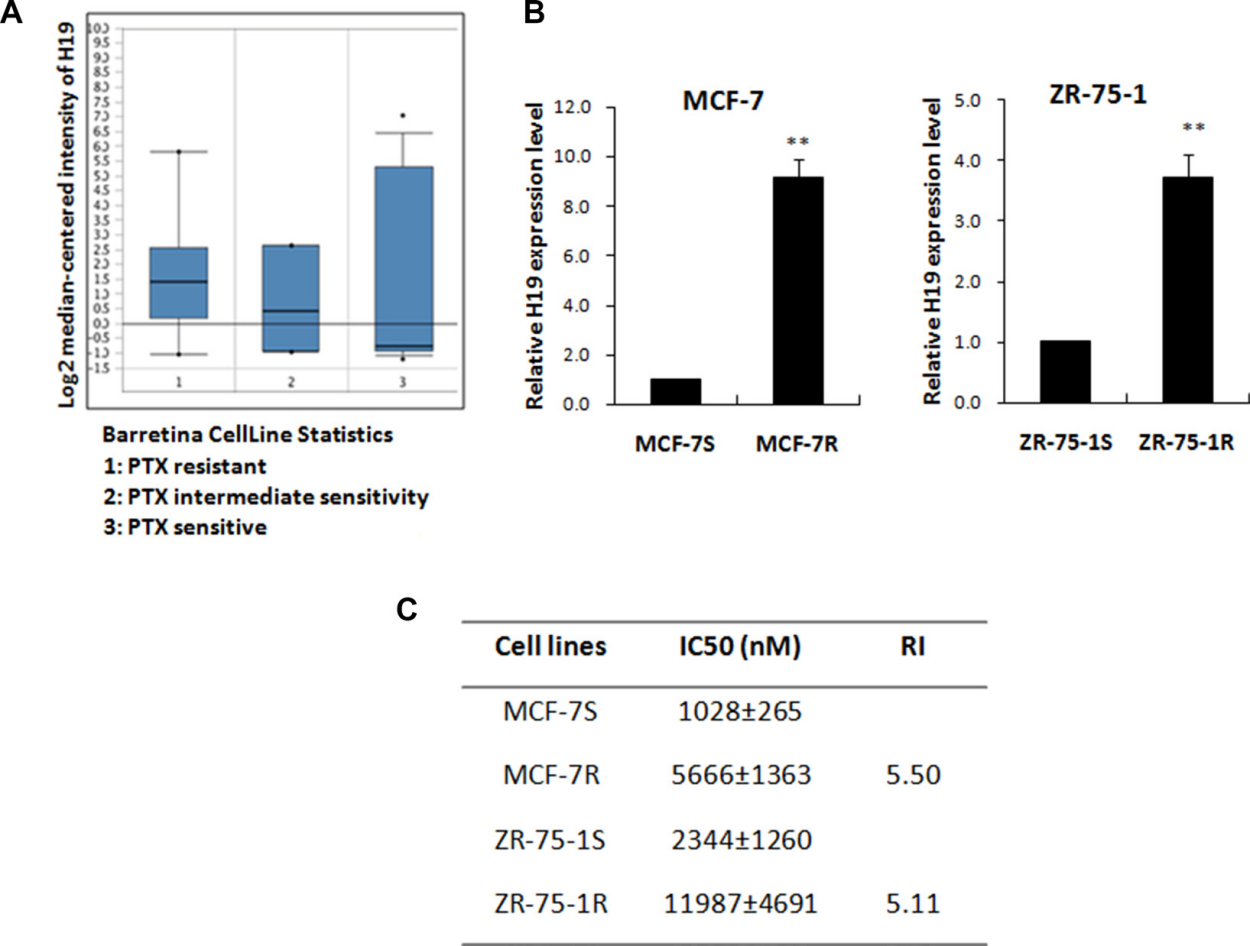
LncRNA H19 was positively correlated with PTX resistance (**A**) The expression level of H19 in breast cancer cells with different degrees of sensitivity to PTX (based on an Oncomine search). The group of cells resistant to PTX consisted of HCC1569, Hs578T, MCF7, MDA-MB-157, MDA-MB-175-VII, MDA-MB-415, MDA-MB-468, and T-47D. The intermediate sensitivity cell group included ZR-75-30, MDA-MB-436, BT-474, and CAL-85-1. The PTX sensitive group consisted of HCC1395, SK-BR-3, CAMA-1, HCC1954, HCC1187, EFM-19, MB157, HMC-1-8, AU565, BT-549, HCC70, HCC1806, BT-20, HDQ-P1, etc. The sensitivity of these cells to PTX was identified by 8-point dose response curves [26]. (**B**) Real-time PCR analysis of H19 expression level in two pairs of PTX-resistant breast cancer cell lines (MCF-7R and ZR-75-1R) and their paired parental cell lines. (**C**) The IC50 values of each pair of PTX-resistant cells.

The expression of lncRNA H19 has been reported in various cancers, where H19 is thought to take part in tumorigenesis and metastasis [23–25]. For this reason, the high expression of H19 in PTX-resistant breast cancer cells attracted our attention. The Oncomine platform (http://www.oncomine.org) is a free online bioinformatics resource of cancer transcriptome data. We analyzed data from Barretina's research, filtered from the Oncomine databases [26], and determined that the expression of H19 increased in parallel with growing resistance to PTX in different breast cancer cell lines (Figure 1A). Real-time PCR confirmed that the H19 RNA level was increased in the PTX-resistant MCF-7 and ZR-75-1 cells (MCF-7R and ZR-75-1R). In Figure 1B, H19 showed dramatically upregulation in MCF-7R and ZR-75-1R cells when compared with the parental MCF-7S and ZR-75-1S cells. However, no upregulation of lncRNA H19 was observed in MDA-MB-231 resistant cells (Data not shown), which are ERα-negative breast cancer cells. The table shows the IC50 for each pair of cell types (Figure 1C). A positive correlation was evident between H19 transcription and PTX resistance in the ERα-positive breast cancer cells.

### LncRNA H19 contributed to drug resistance in breast cancer

**Figure 2:**
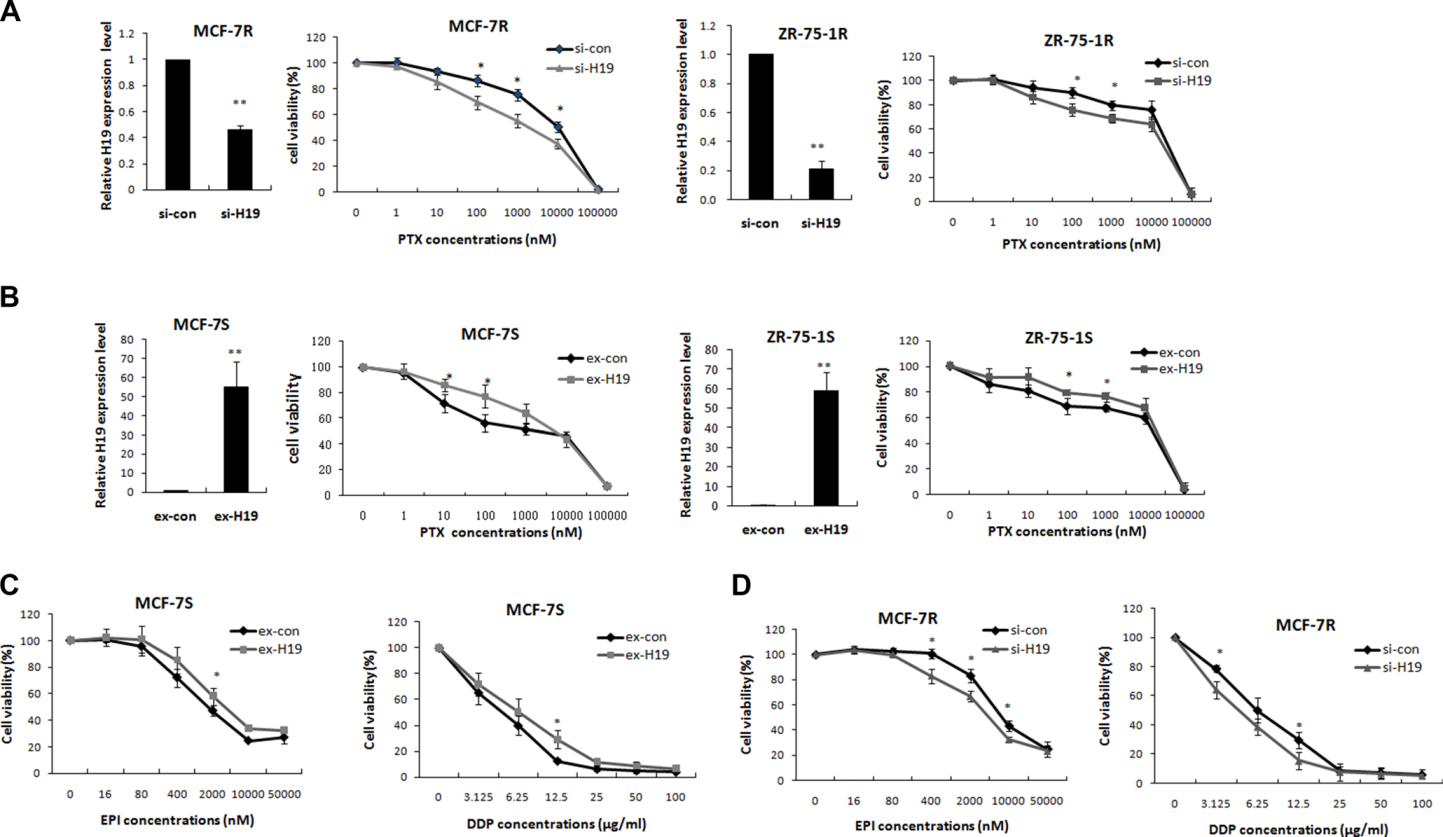
LncRNA H19 contributed to multidrug resistance in breast cancer cells (**A**) MCF-7R or ZR-75-1R cells were transiently transfected with H19-targeting siRNA or scrambled siRNA. Real-time PCR analysis showed that H19 expression was effectively reduced. An MTT assay indicated that knockdown of H19 decreased cell viability under the stress of PTX treatment. (**B**) MCF-7S or ZR-75-1S cells were transiently transfected with H19 overexpression plasmids or control vectors. Real-time PCR analysis confirmed the overexpression of H19. An MTT assay was performed to determine cell viabilities of MCF-7S or ZR-75-1S cells treated with PTX at different concentrations. (**C**) MTT results obtained from MCF-7S cells transfected with H19 overexpression plasmids or control vectors showed that H19 overexpression also increased the resistance of these cells to both EPI (left) and DDP (right). (**D**) The MTT results obtained from MCF-7R cells transfected with H19-targeting siRNA or control siRNA showed that H19 knockdown also decreased the resistance of these cells to both EPI and DDP.

The contribution of lncRNA H19 to PTX resistance in breast cancer was verified by transfecting the MCF-7R and ZR-75-1R cells with H19-targeting siRNA. The interference efficiency was confirmed by real-time PCR (Figure 2A). These transfected cells were treated with a series of PTX concentrations for 48 h, and then harvested for viability tests using the MTT assay. The MCF-7R cells showed markedly decreased viability when treated with 100, 1000, and 10000 nM PTX (Figure 2A). The IC50 values for the MCF-7R cells were reduced from 4172 ± 567 nM to 982 ± 289 nM in the H19 knockdown group. The results were similar for the ZR-75-1R cells, which showed a decrease in the IC50 values from 12791 ± 4703 nM to 5189 ± 3153 nM (Supplementary Table S1).

We further evaluated the role of H19 in breast cancer chemoresistance by ectopic expression of H19 in the parental cell lines MCF-7S and ZR-75-1S. The transfection efficiency was confirmed by qPCR (Figure 2B). At 24 h after transfection, the cells were treated with PTX at different concentrations for 48 h, and then their viability was assessed by the MTT assay. Cells that expressed higher levels of lncRNA H19 showed resistance to PTX treatment. This phenomenon was observed in both MCF-7S and ZR-75-1S cell lines (Figure 2B). The IC50 values increased from 684 ± 201 nM to 2313 ± 817 nM in MCF-7S cells, and from 2563 ± 1358 nM to 6693 ± 2781 nM in ZR-75-1S cells (Supplementary Table S1).

H19 has been reported to induce P-glycoprotein expression under hypoxic conditions [27]. P-glycoprotein, a transmembrane protein encoded by the MDR-1 gene, functions as a pump that promotes the efflux of specific drugs, including PTX. We determined whether the H19-related drug resistance was dependent on P-glycoprotein by using the MTT assay to test the drug response of MCF-7R to two other chemotherapy agents, epirubicin (EPI) and cisplatin (DDP). EPI is another P-glycoprotein substrate, but DDP is not. Introduction of H19 into MCF-7S cells significantly increased their resistance to both EPI and DDP, whereas H19 knockdown attenuated their resistance to both EPI and DDP (Figure 2C and 2D), in accordance with the PTX results. The IC50 values were shown in Supplementary Tables S2 and S3. Cell survival following the stress of chemotherapy drug exposure was therefore promoted by H19 independent of any P-glycoprotein-mediated drug efflux from the cells.

### H19 attenuated the apoptosis response by inhibiting transcription of BIK and NOXA

**Figure 3:**
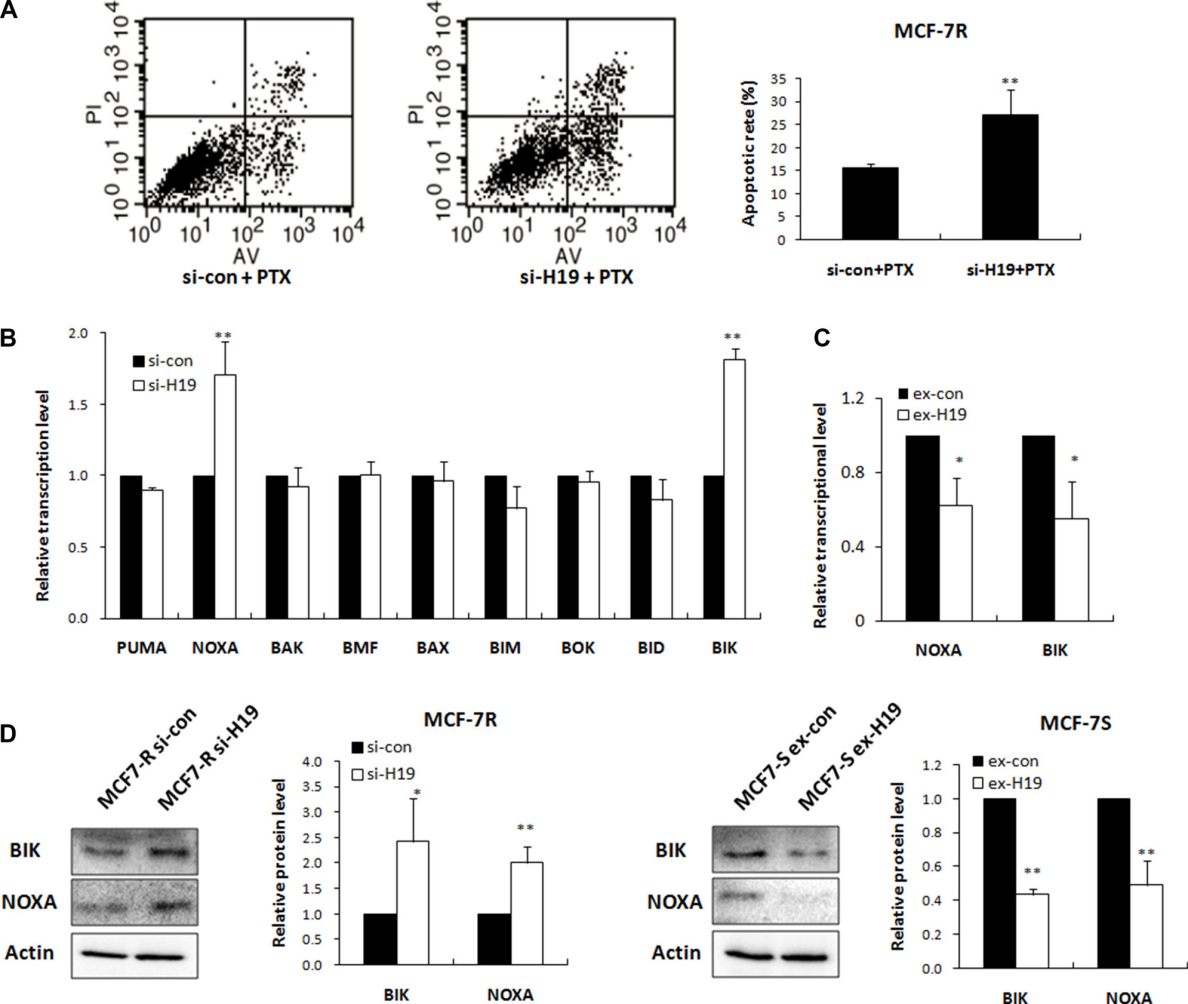
H19 attenuated apoptosis response through inhibiting transcription of BIK and NOXA (**A**) MCF-7R cells were transiently transfected with H19 siRNA or scrambled siRNA, followed by PTX treatment. The apoptosis rates were determined by FACS analysis. (**B**) Real-time PCR analysis was performed to detect the effect of H19 knockdown in MCF-7R cells on mRNA levels of 9 pro-apoptotic genes relative to scrambled control. (**C**) Real-time PCR analysis was performed to detect the effect of H19 overexpression in MCF-7S cells on NOXA and BIK mRNA levels. (**D**) Western blot analysis was performed to detect the effect of H19 overexpression or knockdown on the protein levels of BIK or NOXA.

Most chemotherapy agents exert their effects by activating apoptosis. We evaluated the involvement of apoptosis in H19 mediated chemoresistance by flow cytometry of H19 knockdown cells. The apoptosis rates of MCF-7R cells transfected with H19 siRNA and treated with PTX were evaluated by Annexin V/PI staining and FACS analysis. The H19 knockdown notably increased the apoptotic ratio of the cells treated with PTX (Figure 3A).

We further investigated the mechanism of H19 mediated apoptosis by selecting nine pro-apoptotic genes in the Bcl2 family as the hypothetical H19 targets. As shown in Figure 3B, knockdown of H19 in MCF-7R cells notably upregulated the BIK and NOXA at the transcriptional level, while introduction of H19 into MCF-7S cells significantly suppressed BIK and NOXA expression (Figure 3C). The protein levels of BIK and NOXA were similarly elevated by H19 knockdown and decreased by H19 overexpression (Figure 3D).

**Figure 4:**
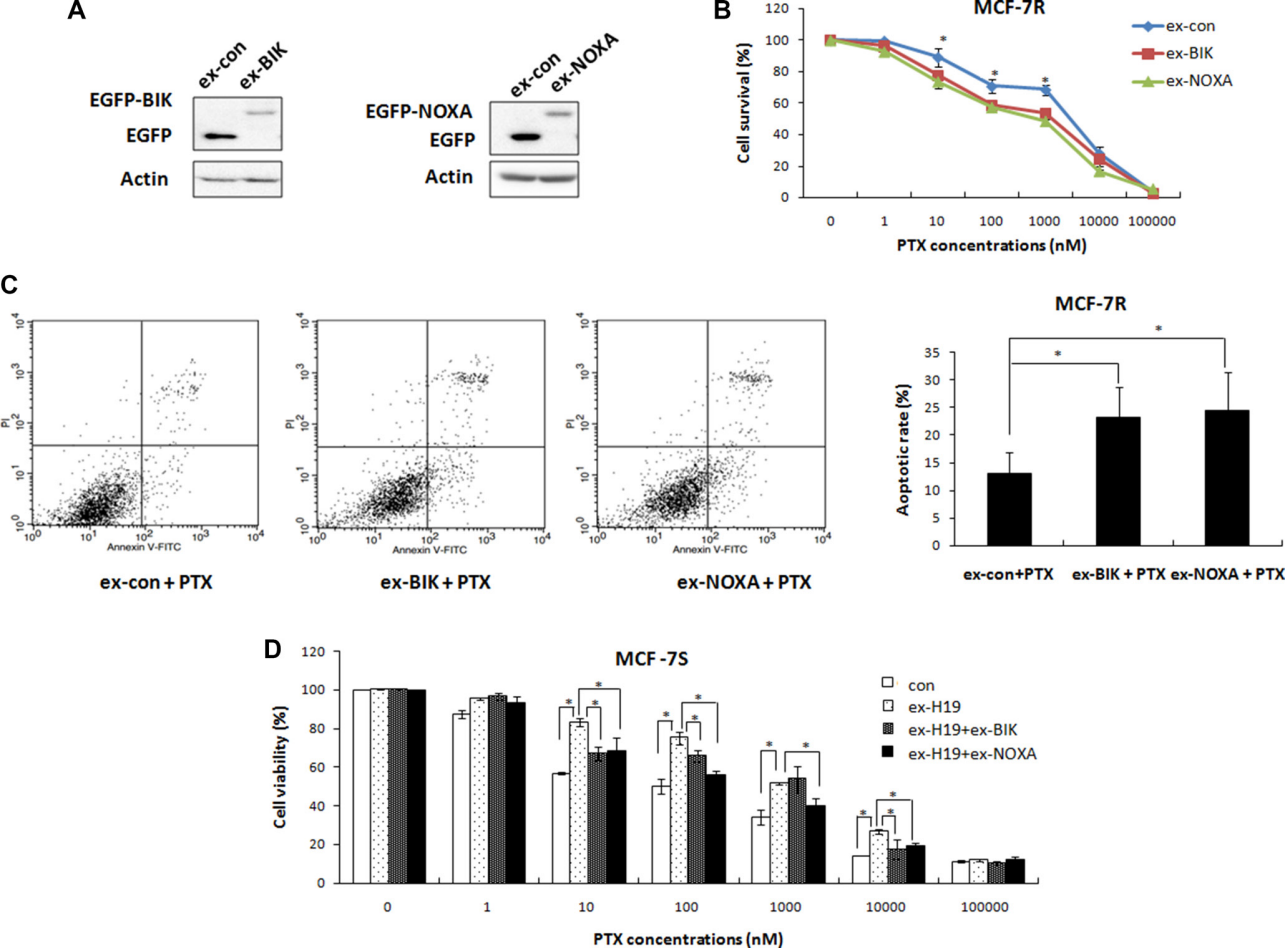
Both BIK and NOXA were involved in H19-mediated drug resistance pathway (**A**) MCF-7R cells were transiently transfected with a BIK (pEGFP-BIK) or a NOXA (pEGFP-NOXA) expression vector, or a control vector. Western blot analysis was performed to detect the expression levels of BIK or NOXA. (**B**) MTT assays performed on these transfected MCF-7R cells showed that overexpression of BIK or NOXA significantly enhanced the sensitivity of these cells to PTX. (**C**) MCF-7R cells transfected with BIK or NOXA expression vectors or control vectors were treated with 200 nM PTX and subsequently harvested for apoptosis analysis. (**D**) MCF-7S cells were transfected with H19 expression plasmids together with either BIK overexpression plasmids or NOXA overexpression plasmids. The cells were treated with PTX at different concentrations and their viability was tested with the MTT assay. BIK overexpression or NOXA overexpression reduced H19-mediated drug resistance.

Following this confirmation of BIK and NOXA as targets of H19, we evaluated their roles in the drug resistance of breast cancer cells by restoring the expression of BIK and NOXA in MCF-7R cells. The transfection efficiency was confirmed by Western blot analysis (Figure 4A). As expected, overexpression of BIK or NOXA decreased cell survival in the presence of PTX, endowing the MCF-7R cells with higher drug sensitivity (Figure 4B). Overexpression of BIK or NOXA also enhanced apoptosis in the MCF-7R cells in response to PTX treatment (Figure 4C), indicating that BIK or NOXA could attenuate the drug resistance phenotype of MCF-7R cells.

We further addressed whether BIK and NOXA were downstream molecules of H19 by co-transfecting H19 and BIK or NOXA expression plasmids into MCF-7S cells. Overexpression of BIK partly restrained the effect of H19 overexpression and sensitized MCF-7S cells to PTX (Figure 4D). A similar result was observed in MCF-7S cells co-transfected with H19 and NOXA expression vectors (Figure 4D). These overexpression and knockdown studies all validated BIK and NOXA as important downstream targets of H19, and confirmed a strong involvement of both genes in the H19-mediated PTX resistance of breast cancer cells.

### H19 was responsible for the epigenetic silencing of BIK in an EZH2 dependent manner

**Figure 5:**
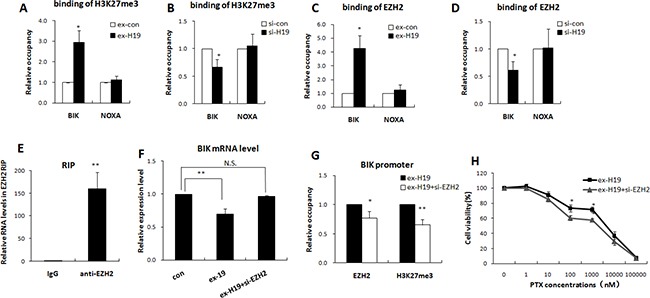
H19 epigenetically silenced BIK in an EZH2 dependent manner (**A** and **B**) Quantitative ChIP analysis of cells transfected with H19 expression plasmids or H19-targeting siRNA showed that altered expression of H19 changed the H3K27me3 modification of the BIK promoter, but not of the NOXA promoter. (**C** and **D**) Quantitative ChIP analysis obtained from cells these transfected cells showed that altered expression of H19 changed the association of EZH2 with the BIK promoter, but not with the NOXA promoter. (**E**) RIP experiments were performed in MCF-7R cells and the co-precipitated RNA was subjected to qRT-PCR for H19. The fold enrichment of H19 in EZH2 RIP is expressed relative to its matching IgG control RIP. (**F**) Real-time PCR analysis showed that the BIK mRNA level was decreased in MCF-7S cells transfected with H19 expression plasmids, while EZH2 knockdown significantly restrained the H19-induced downregulation of BIK. (**G**) MCF-7S cells were transfected with H19 expression plasmids together with EZH2 targeting siRNA. Quantitative ChIP analysis showed that EZH2 knockdown restrained the H19-mediated association of EZH2 and H3K27me3 with the BIK promoter. (**H**) MTT results obtained from these transfected cells showed that knockdown of EZH2 reduced the effect of H19 overexpression on the drug resistance of the MCF-7S cells.

We previously confirmed that H19 inhibits transcription of BIK and NOXA in breast cancer, however, the underlying mechanism is still unknown. A previous study reported that lncRNA H19 suppressed the expression of the target gene by associating with EZH2 in bladder cancer [25]. The polycomb protein EZH2 is a histone methyltransferase that generates trimethylation at lysine 27 of histone H3. A high level of H3K27me3 in the promoter region is recognized as an epigenetic marker indicating the suppression of gene transcription. The modification status of H3K27me3 at the BIK and NOXA promoter regions was therefore checked with ChIP assay to confirm that H19 was responsible for the epigenetic silencing. As shown in Figure 5A and 5B, the BIK promoter region showed a dynamic modification pattern in response to the alteration of H19 expression. High expression of H19 facilitated H3K27me3 modification of the BIK promoter region rather than the NOXA promoter in MCF-7 cells.

We then confirmed the requirement for H19 in EZH2 occupancy of the NOXA and BIK promoters by Q-ChIP analysis of H19-overexpressing MCF-7S cells. As shown in Figure 5C, EZH2 binding onto the BIK promoter was higher when H19 was abundant. By contrast, overexpression of H19 did not increase the occupancy of EZH2 on the NOXA promoter. Corresponding results were observed in H19 knockdown MCF-7R cells. The attenuation of H19 expression reduced the occupancy by EZH2 on the BIK, but not the NOXA, promoter (Figure 5D). We also confirmed that H19 bound to EZH2 in breast cancer cells. As shown in Figure 5E, the endogenous H19 was enriched in the anti-EZH2 RNA immunoprecipitation (RIP) fraction, but showed no enrichment in the IgG fraction.

We further investigated the EZH2 dependence of epigenetic inactivation of BIK by lncRNA H19 by examining whether EZH2 could reverse the H19-induced BIK inactivation. Introduction of H19 into MCF-7S cells significantly decreased BIK expression, while EZH2 knockdown abrogated the effect of H19-induced BIK inhibition (Figure 5F). The H19-induced upregulation of EZH2 binding occupancy and the H3K27me3 modification at the BIK promoter region were reversed by concomitant knockdown of EZH2 (Figure 5G).

We then confirmed that the H19-BIK mediated chemoresistance was dependent on EZH2 by transfecting MCF-7S cells with H19 expression plasmids, together with EZH2 targeting siRNAs, and treating these cells with different concentrations of PTX. MTT assays showed that EZH2 knockdown partly restrained the effect of H19 and sensitized the MCF-7S cells to PTX (Figure 5H). Taken together, these results revealed that epigenetic silencing of BIK by H19 was dependent on EZH2.

### H19 mediated ERα-induced PTX resistance in breast cancer

**Figure 6:**
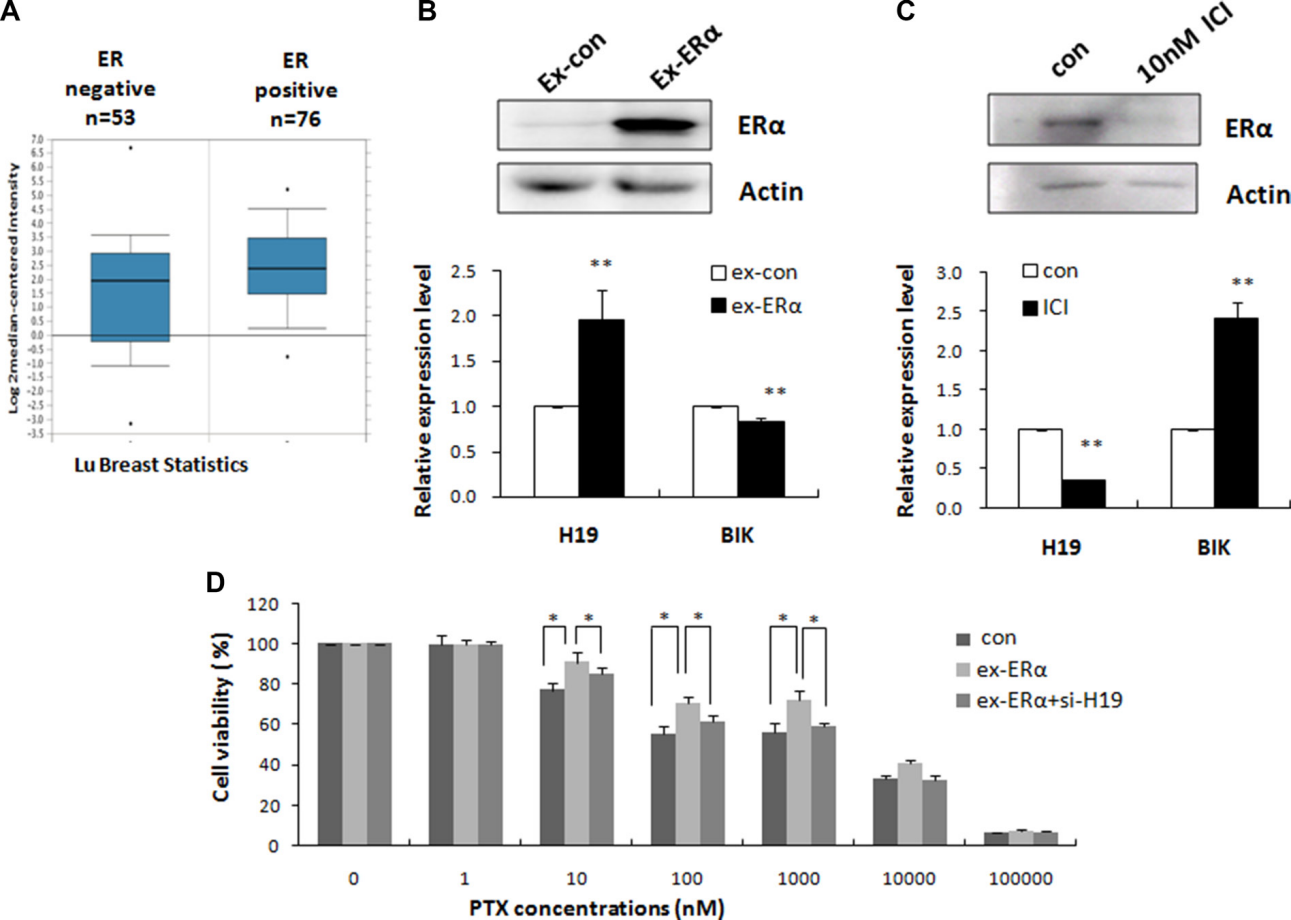
H19 mediated ERα-induced PTX resistance in breast cancer (**A**) H19 expression levels in tumor tissues from patients with ER-positive and ER-negative breast cancers (Oncomine). (**B**) MCF-7S cells were transiently transfected with ERα expression vector or control vector. Western blot analysis was performed to detect the expression level of ERα (upper). Real-time PCR was performed to detect the mRNA levels of H19 and BIK (lower). (**C**) Western blot analysis was performed to detect the expression level of ERα in MCF-7R cells treated with ERα inhibitor (upper). Real-time PCR was performed to detect the mRNA levels of H19 and BIK (lower). (**D**) MCF-7S cells were transfected with ERα expression plasmids, or ERα expression plasmids together with H19 targeting siRNA. MTT assay showed that H19 knockdown reduced the effect of ERα overexpression on the drug resistance of the MCF-7S cells.

Elevated expression of H19 was only observed in ERα-positive PTX-resistant breast cancer cell lines (MCF-7 and ZR-75-1) in our study; therefore, we proposed that ERα could activate H19 expression. We evaluated the relationship between ERα and H19 by analyzing published patient data using Oncomine. Lu's breast cancer dataset with 129 samples [28] showed a higher expression of H19 in ERα-positive (*n* = 76) than ERα-negative (*n* = 53) breast cancers (Figure 6A), which suggested a possible involvement of ERα in the upregulation of H19 in chemoresistant cancer cells. Overexpression of ERα in MCF-7S upregulated H19 and down-regulated BIK transcription (Figure 6B). Conversely, inhibition of ERα by the ERα inhibitor ICI significantly decreased H19 and increased BIK expression (Figure 6C).

ERα is reportedly a powerful chemoresistance factor [29]. We therefore examined the possible involvement of H19 in ERα-mediated drug resistance pathways. Specifically, we tested whether knockdown of H19 could rescue ERα-induced drug resistance. As shown in Figure 6D, suppression of H19 expression restrained the effect of overexpression of ERα and sensitized MCF-7S cells to PTX. These results, taken together, indicated that ERα promoted H19 expression in breast cancer cells and supported H19 as an important mediator of ERα-induced drug resistance.

## DISCUSSION

Acquisition of drug resistance is one of the main obstacles preventing successful cancer therapy. Previous studies investigating the molecular basis of chemoresistance have tended to focus on coding genes and the functions of their protein products. However, recent research is now increasingly emphasizing the importance of lncRNAs as integral components of gene regulatory networks. Therefore, more studies are needed to elucidate the potential roles of lncRNA in drug resistance. Our findings in the present study indicate that high expression of lncRNA H19 may reduce the sensitivity of breast cancer cells to chemotherapy through inactivation of pro-apoptosis pathways.

Cell apoptosis is the most commonly activated pathway during chemotherapy. Consequently, disruption of apoptosis facilitates multidrug resistance. The Bcl2 family members are the key regulators of cell apoptosis. We identified BIK and NOXA, two members of the Bcl2 family, as targets of H19 (Figure 3B), by showing that ectopic expression of BIK or NOXA reversed H19-mediated PTX resistance (Figure 4D). Both BIK and NOXA are BH3 only pro-apoptotic proteins located on the outer mitochondrial membrane and appear to be critical effectors of apoptosis [30, 31]. Inhibition of BIK and NOXA by H19 reduced apoptosis in breast cancer cells and their subsequent sensitivity to drugs. A previous study reported that blocking H19 expression induced cell apoptosis [15], indicating that H19 is a direct regulator of apoptosis pathways. Besides that, H19 related chemoresistance in hepatocellular carcinoma cells was associated with induction of MDR1 [14]. However, in the present study, knockdown of H19 significantly reversed resistance even to drugs that were not substrates of P-glycoprotein (Figure 2D), indicating H19 induced breast cancer chemoresistance through regulation of basic cellular activities rather than by drug efflux.

Epigenetic alterations are a hallmark of cancer, and aberrant histone modification patterns lead to abnormal silencing of tumor suppressor genes. EZH2 is a critical component of Polycomb-Repressive Complex 2 (PRC2), which is a type of histone methyltransferase (HMT). EZH2 is responsible for generating histone H3 lysine 27 trimethylation, a modification that always correlates with transcriptionally repressed chromatin. Some lncRNAs have been reported to recruit PRC2 complexes to specific loci and repress target gene expression [32–34]. In the present study, we demonstrated that H19 could recruit EZH2, catalyze trimethylation of H3K27 in the promoter region of BIK, and suppress gene transcription.

BIK, a member of the Bcl2 family, is a tumor suppressor, and its expression is prevented by chromosomal deletions encompassing the BIK locus or by DNA methylation in several human cancers [35–37]. We revealed that BIK could also be silenced by H3K27me3 histone modification induced by H19 and EZH2. Restoration of BIK expression by removal of epigenetic markers via inhibition of these lncRNAs or by DNA methyltransferases (DNMTs) / Histone methyltransferases (HMTs) inhibitors could therefore represent a potential therapeutic strategy for treatment of refractory breast cancer.

The molecular mechanism of lncRNA-mediated transcriptional regulation is complex. LncRNAs can downregulate gene expression in a variety of ways, including the establishment of repressive chromatin states by associations with HMTs or DNMTs, thereby changing histone modification and DNA methylation, and by regulation of target mRNA stability or translational efficiency through RNA-RNA interactions [38–41]. NOXA, another target of lncRNA H19, also encodes a pro-apoptosis protein of the Bcl2 family, but we found no repression of NOXA transcription due to H19-induced H3K27me3 modification. We propose that H19 inhibits NOXA transcription by other not yet identified mechanisms. Further work is needed to determine the reason for down-regulation of NOXA in breast cancer chemoresistance.

Aberrant H19 expression in breast cancer could arise through various mechanisms, including chromosomal abnormalities, transcription factor binding, and epigenetic alterations. Here, we found that H19 was upregulated by ERα. Previous reports have shown that ER-positive tumors were more vulnerable to resistance to chemotherapy agents. Several mechanisms have been proposed to explain ERα-mediated drug resistance, including inhibition of apoptosis and enhanced P-glycoprotein expression [21, 42]. Previous research has even determined that BIK mRNA and protein were strongly induced by estrogen starvation or fulvestrant treatments [43]. We propose that the ERα-H19-BIK axis may be a novel therapeutic target for treatment of patients with ERα-positive, chemoresistant breast cancer. Despite the identification of ERα as the upsteam molecular of H19, we also concerned the expression of ERβ and progesterone receptor (PR) in chemoresistant breast cancer cells. Western blot analysis showed the expression of ERβ was not changed in MCF-7R compared with MCF-7S cells, while PR was up regulated in drug-resistant cells (Supplementary Figure S1A and S1B). It suggested PR might also be involved in breast cancer chemoresistance in related to H19. It will be very interesting to investigate the potential contribution of PR to the transcriptional regulation of lncRNA H19 further.

The aim of the present study was to identify new mechanisms underlying chemoresistance in order to lay the foundation for therapies that could have greater efficacy. Collectively, we showed that H19 was an important factor contributing to drug resistance in breast cancer, and that epigenetic regulation mediated by H19 and EZH2 participates in the acquisition of chemoresistance. Therefore, therapies that target the regulatory signaling axis ERα-H19-BIK may increase the overall effectiveness of breast cancer chemotherapy.

## MATERIALS AND METHODS

### Cell culture

Human MCF-7 and ZR-75-1 breast cancer cell lines were obtained from ATCC. The PTX-resistant MCF-7R and ZR-75-1R cell lines were established by pulse selection of MCF-7 and ZR-75-1 cells with paclitaxel; the parental cell lines were named MCF-7S and ZR-75-1S. The MCF-7S and MCF-7R cells were cultured in MEM supplemented with 10% FBS, insulin (0.2 U/ml), 100 U/ml penicillin, and 100 U/ml streptomycin, whereas ZR-75-1S and ZR-75-1R cells were cultured in DMEM containing 10% FBS, 100 U/ml penicillin, and 100 U/ml streptomycin.

### Transfection of breast cancer cells

Breast cancer cells were transfected with siRNA oligonucleotides or plasmids using Lipofectamine 2000 (Invitrogen), according to the manufacturer's protocol. The nucleotide sequence of siRNA for H19 was CCAACAUCAAAGACACCAU and for EZH2 was GAGGUUCAGACGAGCUGAUUU. The siRNAs for the target genes and negative control (si-con) were purchased from Invitrogen. The ERα expression vector was described in our previous work [42]. The BIK and the NOXA expression vectors containing their respective full length coding sequences were cloned into a pEGFP-N3 vector for expression of EGFP-BIK or EGFP-NOXA fusion proteins. The H19 expression vector was cloned into pcDNA3.0.

### RNA extraction and qRT-PCR assay

Total RNA was extracted using Trizol reagent (Invitrogen), according to the manufacturer's protocol. The cDNA was prepared by reverse-transcribing 1 μg of total RNA, according to the manufacturer's instructions (Vazyme). The sequences of primers designed specifically for real-time PCR were as follows: H19-F, ATCGGTGCCTCAGCGTTCGG; H19-R, CTGTCCTC GCCGTCACACCG; PUMA-F, ATGGCGGACGACCT CAAC; PUMA-R, AGTCCCATGAAGAGATTGTAC ATGAC; NOXA-F, GCAGAGCTGGAAGTCGAGTGT; NOXA-R, CTCTTTTGAAGGAGTCCCCTCAT; BAK-F, GCTCCCAACCCATTCACTAC; BAK-R, TCCCTACTC CTTTTCCCTGA; BMF-F, CCACCAGCCAGGAAGAC AAAG; BAX-F, TGGAGCTGCAGAGGATGATTG; BAX-R, GAAGTTGCCGTCAGAAAACATG; BIM-F, AGCCGAAGACCACCCACGAA; BIM-R, GCTCCCTC CTTTACATTCACAACAA; BOK-F, GGCCCAGCGTC TACCGCAA; BOK -R, CGCATACAGGGACACCACCT; BID-F, CCATAAGGAGGAAGCGGGTAG; BID-R, CG TTGTTGACCTCACAGTCCA; BIK-F, GACCATGGAG GTTCTTGGCA; BIK-R, AGGCTCACGTCCATCTCGTC.

### Western blot assay

Total protein extracts were obtained by lying cells in ice-cold lysis buffer. The proteins were separated on SDS-polyacrylamide gels and transferred to PVDF membranes (Bio-Rad). After blocking in 5% skimmed milk for 1 h, the membranes were incubated with a primary antibody overnight at 4°C. Membranes were washed 3 times for 10 min in TBST and incubated with an HRP-conjugated secondary antibody for 1 h at room temperature. After washing 3 times for 10 min in TBST, the proteins were visualized with an ECL detection system. The EZH2 antibody was purchased from Millipore, the ERα antibody was from Santa Cruz, and the β-actin antibody was obtained from Sigma-Aldrich.

### Survival curves

Cells were seeded at a density of 8000 cells per well in 96-well plates. On the following day, cells were treated with graded concentrations of PTX, EPI, or DDP for 48 h. At the end of the culture, cell viability was measured using the MTT assay as previously described [44]. All measurements were done in triplicate.

### Apoptosis assay

An apoptosis detection kit (Keygentech) was used for apoptosis analysis, following the manufacturer's instructions. Briefly, cells were collected, washed twice with PBS, and gently resuspended in 500 μl binding buffer. After addition of 5 μl Annexin V-FITC and 5 μl propidium iodide (PI), the cells were incubated in the dark for 15 min at room temperature, followed by immediate flow cytometry analysis on a FACScan instrument (Becton Dickinson).

### Chromatin immunoprecipitation (ChIP) assay

Chromatin immunoprecipitation (ChIP) assays were performed using the ChIP assay kit, essentially as described in the manufacturer (Millipore). Briefly, 1 × 10^7^ cells were fixed in 1% formaldehyde at 37°C for 10 min. The cells were then lysed, sonicated to generate 200–1000 bp fragments, and incubated with antibody overnight at 4°C. Reversal of cross-linking was carried out at 65°C for 5 h, followed by DNA isolation. Quantitative analysis of the ChIP products was carried out by real-time PCR using the SYBR Green real-time PCR Master Mix (Vazyme) according to the provided instructions. The EZH2 antibody was from Abcam and the H3K27me3 antibody was from Millipore. The sequences for qChIP were as follows: NOXA-F, GTTGCCTAAGGTTTGTAGCCAG; NOXA-R, TCCAGGCTCATTTTGACTTACC; BIK-F, CAAGCTTGCAGAACAGCAGG; BIK-R, TGGCATTGG CAACAGAACC.

### RNA immunoprecipitation (RIP) assay

The RIP experiments were performed using a Magna RIP™ RNA-Binding Protein Immunoprecipitation Kit (Millipore), according to the manufacturer's instructions. The EZH2 antibody was obtained from Abcam. The co-precipitated RNAs were detected by reverse-transcription PCR. The total RNAs were the input controls.

### Statistical analysis

All experiments were repeated three times. The statistical differences between means were determined with a *t*-test. Values are expressed as means ± standard error of triplicate measurements. *P* < 0.05 was considered statistically significant.

## SUPPLEMENTARY MATERIALS


