# Supplementary material for: LncRNA H19 confers chemoresistance in ERα-positive breast cancer through epigenetic silencing of the pro-apoptotic gene BIK
Source: Oncotarget. 2016 Nov 10;7(49):81452–62. doi: 10.18632/oncotarget.13263 (PMC5348405; doi:10.18632/oncotarget.13263)
Supplement: Supplementary file 1 [file oncotarget-07-81452-s001.pdf]

# LncRNA H19 confers chemoresistance in ERα-positive breast cancer through epigenetic silencing of the pro-apoptotic gene BIK

## Supplementary Materials

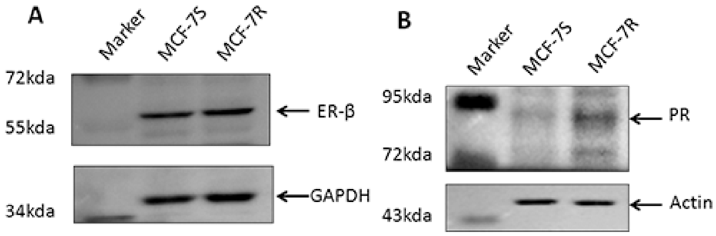


**Supplementary Figure S1: The expression levels of ERβ and PR in MCF-7S and MCF-7R cells.** (**A**) Western blot analysis was performed to detect the protein level of ERβ in MCF-7S and MCF-7R cells. (**B**) Western blot analysis was performed to detect the protein level of PR in MCF-7S and MCF-7R cells.

**Supplementary Table S1: IC50 values were calculated according to the results of MTT in Figure 2A and 2B**

| Cell lines | | IC50 (nM) |
|---|---|---|
| MCF-7R | si-con | 4172 ± 567 |
| | si-H19 | 982 ± 289 |
| ZR-75-1R | si-con | 12791 ± 4703 |
| | si-H19 | 5189 ± 3153 |
| MCF-7S | ex-con | 684 ± 201 |
| | ex-H19 | 2313 ± 817 |
| ZR-75-1S | ex-con | 2563 ± 1358 |
| | ex-H19 | 6693 ± 2781 |

IC50 values were determined using SPSS software and showed as means ± standard error of triplicate measurements.

**Supplementary Table S2: IC50 values were calculated according to the results of MTT in Figure 2C**

| Drug | MCF-7S | IC50 |
|---|---|---|
| EPI | ex-con | 2615 ± 589 nM |
| | ex-H19 | 5897 ± 1896 nM |
| DDP | ex-con | 4.4 ± 1.1 μg/ml |
| | ex-H19 | 6.4 ± 2.0 μg/ml |

IC50 values were determined using SPSS software and showed as means ± standard error of triplicate measurements.

**Supplementary Table S3: IC50 values were calculated according to the results of MTT in Figure 2D**

| Drug | MCF-7R | IC50 |
|---|---|---|
| EPI | si-con | 10660 ± 2886 nM |
| | si-H19 | 5491 ± 870 nM |
| DDP | si-con | 6.94 ± 1.27 μg/ml |
| | si-H19 | 4.32 ± 0.86 μg/ml |

IC50 values were determined using SPSS software and showed as means ± standard error of triplicate measurements.
